# Three-dimensional printing for heart diseases: clinical application review

**DOI:** 10.1007/s42242-021-00125-8

**Published:** 2021-04-30

**Authors:** Yanyan Ma, Peng Ding, Lanlan Li, Yang Liu, Ping Jin, Jiayou Tang, Jian Yang

**Affiliations:** Department of Cardiovascular Surgery, Xijing Hospital, Airforce Medical University, Xi’an, 710032 China

**Keywords:** Three-dimensional printing, Congenital heart disease, Transcatheter aortic valve replacement, Heart diseases, Cardiac imaging techniques

## Abstract

Heart diseases remain the top threat to human health, and the treatment of heart diseases changes with each passing day. Convincing evidence shows that three-dimensional (3D) printing allows for a more precise understanding of the complex anatomy associated with various heart diseases. In addition, 3D-printed models of cardiac diseases may serve as effective educational tools and for hands-on simulation of surgical interventions. We introduce examples of the clinical applications of different types of 3D printing based on specific cases and clinical application scenarios of 3D printing in treating heart diseases. We also discuss the limitations and clinically unmet needs of 3D printing in this context.

## Introduction

Cardiovascular diseases comprise the leading causes of death in all areas of the world and can be categorized as coronary artery disease, rheumatic heart disease, cardiomyopathy, congenital heart disease (CHD), valvular heart disease, carditis, and aortic aneurysms [1, 2]. As the population ages and progress continues in cardiovascular treatment options, cardiac surgeons and interventional cardiologists are confronted with complex and unpredictable anatomical challenges.

Medical three-dimensional (3D) printing, also known as rapid prototyping or additive manufacturing, is the process of converting digital signals into physical models using 3D printers. It enables visual inspection and direct manipulation of models of human anatomy and pathology [3]. It has been nearly 30 years since this technology was introduced into medicine. Three-dimensional printing was first used in orthopedics and maxillofacial surgery, especially in the manufacturing of surgical guides and personalized grafts involving the skull, mandible, dentures, and artificial joints [4–6]. As 3D printing gained a foothold in other medical areas, evidence began to accumulate the usefulness of 3D-printed applications in the treatment of heart diseases, such as CHDs, valvular diseases, and hypertrophic cardiomyopathy [7–9]. This review summarizes cardiac 3D printing workflow and discusses the clinical applications, current status, limitations, and future perspectives of cardiac 3D printing.

## Fundamentals of cardiac three-dimensional printing

**Fig. 1:**
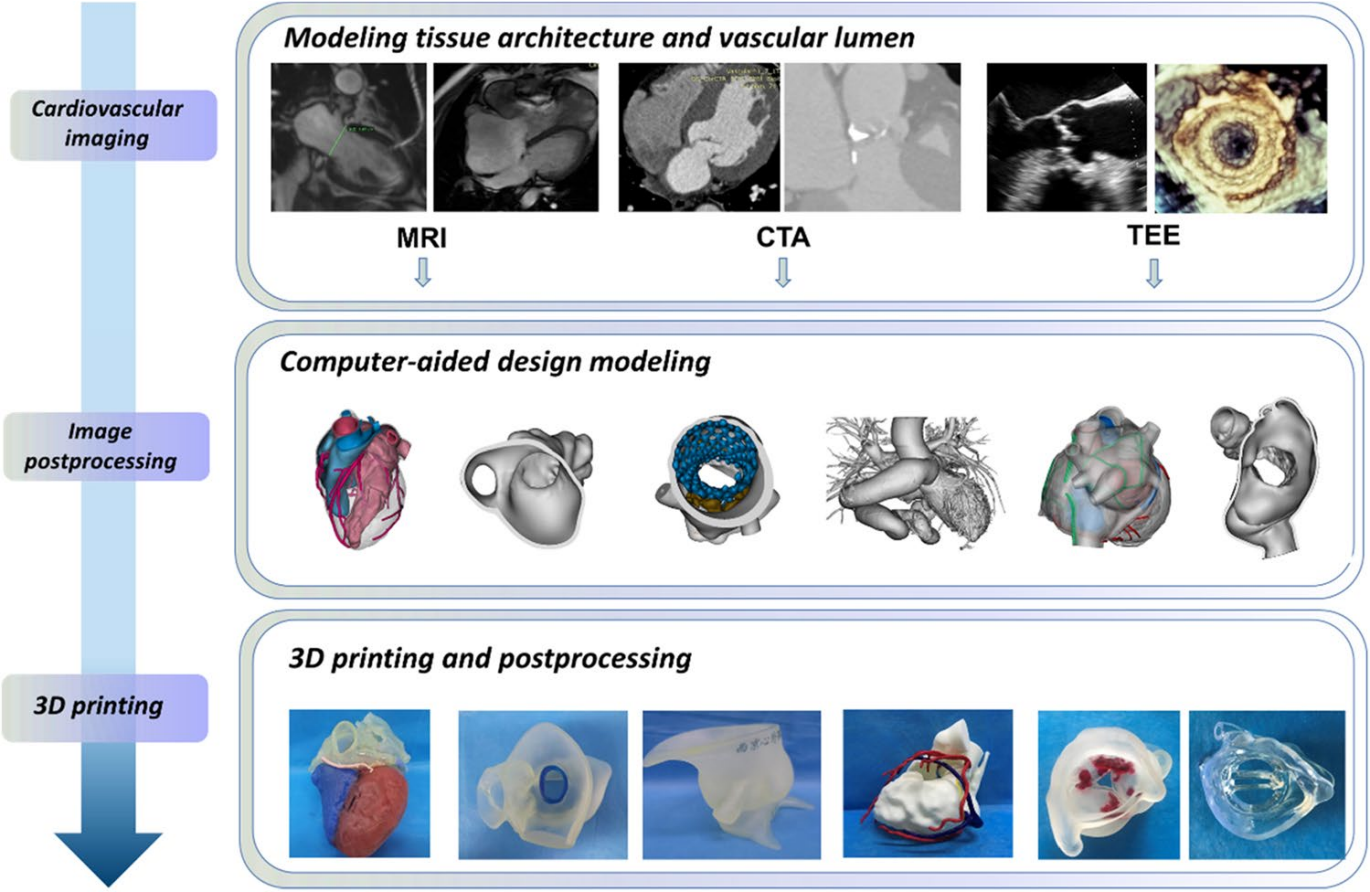
Three-dimensional printing flowchart of the cardiac system. MRI: magnetic resonance imaging, CTA: computed tomography angiography, TEE: transesophageal echocardiography, 3D: three-dimensional

Generating a 3D-printed heart model includes sequential stages of image acquisition, data postprocessing, and industrial-level manufacturing. Acquisition of 3D volumetric cardiovascular images from computed tomography (CT), magnetic resonance imaging (MRI), 3D transthoracic echocardiography, or transesophageal echocardiography is the first step. Among these imaging modalities, CT angiography (CTA) is the one most frequently used, and CT images can be reconstructed ideally at about 1 mm thick. The second step in the 3D printing flowchart is image postprocessing with the aid of commercially available software programs (i.e., Materialise Mimics 21.0, Materialise, Leuven, Belgium), which discriminates between the anatomical area of interest and the adjacent tissues. During this process, DICOM (Digital Imaging and Communications in Medicine) cardiac images from radiology and cardiac imaging workstations are transformed and universally stored in standard tessellation language (STL) format. Computer-aided design software is used to refine the STL file by segmenting, wrapping, smoothing, and augmenting the model, manually or automatically, to exemplify anatomical and pathological areas and by adding connectors between separate anatomical structures of interest before printing. Afterward, a 3D printer (i.e., J750, Stratasys, Eden Prairie, MN, USA) interprets data in an STL file to manufacture a physical object. Material jetting technology, which can combine several materials within the same 3D-printed cast, thereby enabling the fabrication of models of human anatomy and pathology that contain different tissues, is the present ideal 3D printing technique. Finally, postprocessing by removal of support materials, ultraviolet curing, polishing, cleaning, sterilization, and labeling is also crucial to the quality of the models [10–12]. The 3D printing flowchart in the cardiac system is illustrated in Fig. 1.

## Cardiac three-dimensional printing applications

Applications of 3D printing in the cardiac field range from CHDs, valvular heart diseases, left atrial appendage occlusion (LAAO), arrhythmia, to hypertrophic cardiomyopathy (Table 1).

**Table 1:** Three-dimensional printing applications in the cardiac field

Cardiac diseases or procedures	Related centers	References
*CHDs*		
Tetralogy of Fallot TGA DORV DORV with remote VSD DORV with subpulmonary VSD DORV with subaortic VSD HLHS for the Norwood operation TGA combined with VSD and severe pulmonary valve stenosis Cor triatriatum Coarctation or disconnection of aorta PA combined with a single ventricle Vascular ring Persistent truncus arteriosus Mirror-image dextrocardia	Toronto Children's Hospital Westchester Medical Center Hospital Virgen del Rocio Xijing Hospital West China Hospital Xiangya Second Hospital of Central South University	[29–31]
TAVR	Xijing Hospital Georgia Institute of Technology and the Piedmont Heart Institute University of Minnesota University of London and St. George’s University Hospitals	[10, 13, 14]
LAAO	St Vincent’s Public Hospital The First Affiliated Hospital of Xi’an Jiaotong University Hôpital Privé “Les Franciscaines” University of Texas Southwestern Medical Center Prince of Wales Hospital	[41–44]
Hypertrophic cardiomyopathy	Xijing Hospital	[48–50]

CHDs: congenital heart diseases, TGA: transposition of the great arteries, DORV: double outlet right ventricle, VSD: ventricular septal defect, HLHS: hypoplastic left heart syndrome, PA: pulmonary atresia, TAVR: transcatheter aortic valve replacement, LAAO: left atrial appendage occlusion

**Fig. 2:**
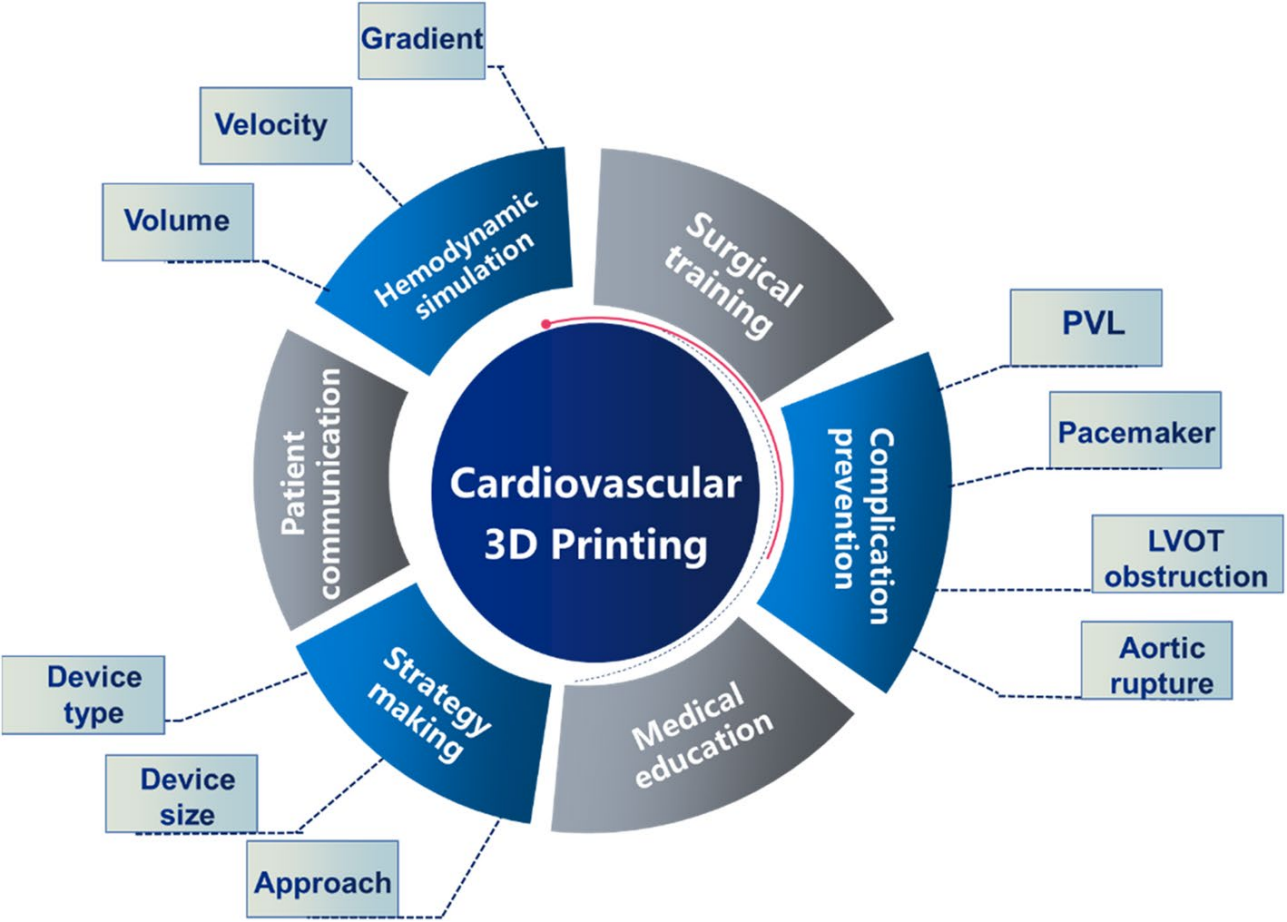
Function of three-dimensional printing in the cardiovascular field. 3D: three-dimensional, PVL: paravalvular leak, LVOT: left ventricular outflow tract

Three-dimensional printing provides unparalleled tactile perception and true volumetric assessment of complex cardiovascular pathological conditions by offering unprecedented illustrations and spatial appreciation of cardiovascular structures. First, it can be used for patient education and doctor–patient communication [15]. In the past, patient educational methods included using paper materials, watching videos, and engaging in patient–doctor dialogue. However, these methods can only target diseases, not individual cases. Moreover, individual differences exist in terms of patients’ understanding of diseases, learning capacity, and compliance. Due to the advantage of 3D printing in facilitating patient education and doctor–patient communication, 3D printing-assisted perioperative educational intervention has been shown to effectively control the anxiety of patients with acute trauma, relieve pain, and improve sleep satisfaction [16]. Second, 3D printing plays a useful role in the study and application of investigational devices. For newly developed cardiac medical devices, using a 3D printing technique can reduce the cost and time of molding, thus optimizing the whole design process. Third, 3D printing acts as an ideal tool for simulating surgery and surgical planning. Cardiovascular physicians can carry out a computer-aided 3D virtual operation based on individualized medical image data to find the best surgical plan for individual patients by simulated analysis, which takes advantage of the characteristics of computer hemodynamics [17]. Through virtual surgical design and computational hemodynamic simulation, 3D printing technology can also be used to perform and predict quantitative changes in hemodynamic parameters and blood flow trajectories during the operation and reduce surgical risks by providing reliable information. One can measure in advance important parameters such as gradient, velocity, and volume using the 3D-printed model [18–20]. One can further use the physical 3D-printed model to practice cutting, stitching, and knotting skills. Meanwhile, strategy development including selection of device type, device size, and different approaches is crucial to the success of cardiovascular intervention; 3D-printed models can provide sufficient information to make appropriate choices [11, 21]. Furthermore, careful planning before the operation or the intervention using the 3D models, including anticipation of complications such as annular rupture, paravalvular leak, the need for a pacemaker, and coronary artery occlusion, can further guarantee procedural success [22]. Lastly, 3D printing technology plays an important role in training medical professionals and students. Because the anatomical structure of the heart is delicate, complicated, and difficult for beginners to comprehend, the lack of satisfactory teaching tools presents an unavoidable obstacle. With the introduction of 3D printing technology, medical professionals can now practice with realistic anatomical models before the operation to improve the success rate and accuracy of the operation [23]. Not only can it help reduce costs, but it can also reduce radiation and procedural times. In particular, it provides an excellent model for teaching medical professionals and medical students to understand the changes that occur in the heart and its connection with surrounding vessels in different disease states [24–26]. The function of 3D printing in the cardiovascular field is illustrated in Fig. 2.

## Three-dimensional printing and congenital heart disease

The incidence of CHDs is 0.8%–1.2%, and most patients with CHD need surgical or interventional treatment. CHD is characterized by a complex anatomical structure and great individual variability. Although dynamic 3D echocardiography, multislice spiral CT, MRI, and other imaging techniques have shown their advantages in the diagnosis of complex CHD, it is still difficult to obtain intuitive anatomical details. By transforming individual imaging data into physical models, 3D printing technology can reproduce the pathological structure and precisely represent the internal and external spatial structures of the heart, thereby providing the basis for an accurate diagnosis [27, 28]. Shi-Joon Yoo of Toronto Children's Hospital reported that 3D-printed models, such as those for a double outlet right ventricle and hypoplastic left heart syndrome, were better than imaging data for preoperative surgical training and evaluation [29]. Harikrishnan et al. at the Westchester Medical Center, New York, printed the heart model of a patient with pulmonary atresia combined with a single ventricle [30]. With the help of this model, a one and half single ventricle corrective procedure was successfully completed. Israel Valverde et al. printed a 3D heart model for a child with transposition of the great arteries, ventricular septal defect, and severe pulmonary valve stenosis [31]. The location and size of the ventricular septal defect and its spatial anatomical structure with the great artery were clearly displayed on the model.

**Fig. 3:**
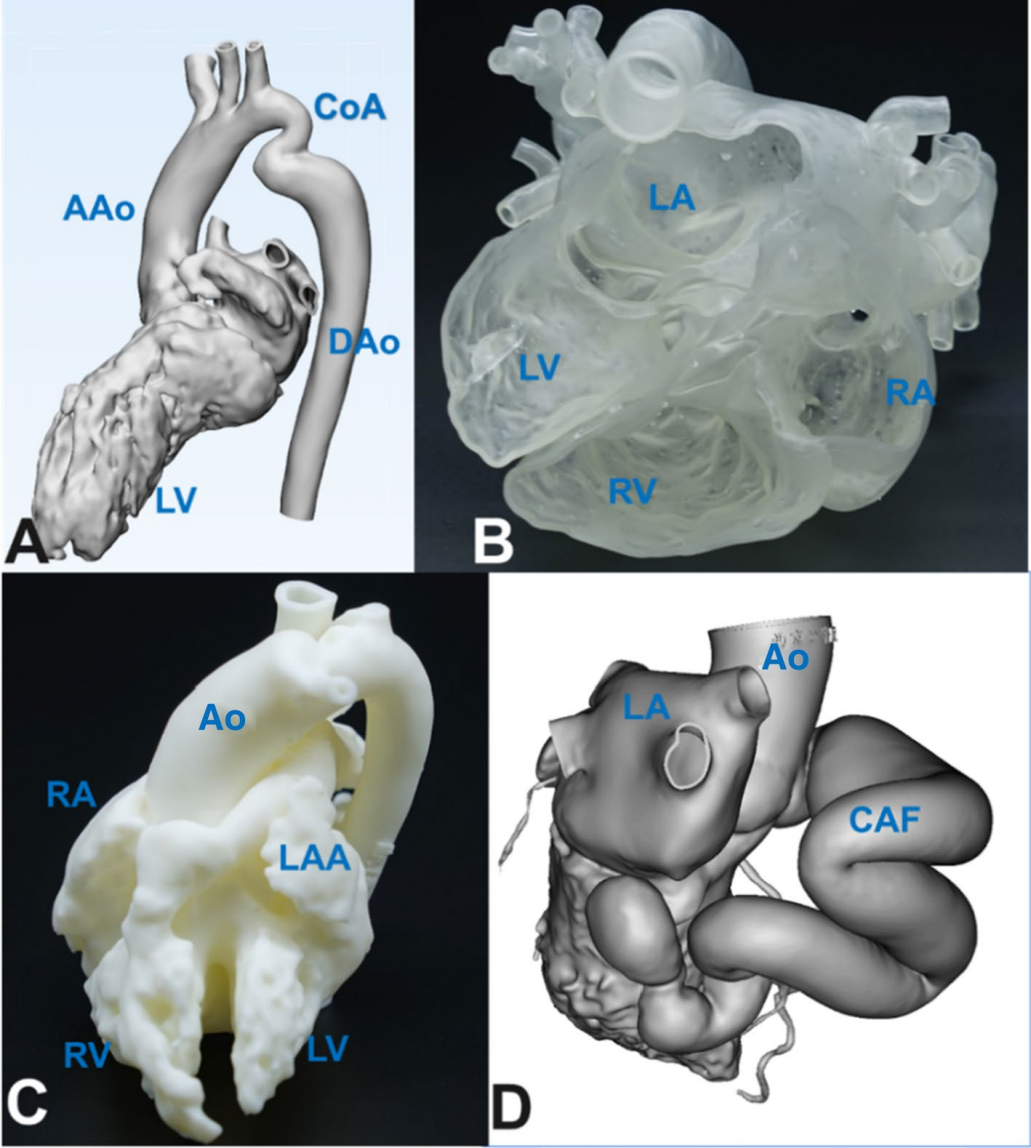
Three-dimensional (3D)-printed model of congenital heart diseases. **a** Computer modeling shows the internal structural profile of complex congenital heart diseases and coarctation of the aorta. The ascending aorta and descending aorta remain normal. **b** 3D-printed model of cor triatriatum. The left atrium of the patient is separated into false and true chambers by a septum; the structures of the left ventricle, the right atrium, and the right ventricle remain normal. **c** 3D-printed model of a double outlet right ventricle. The right ventricle of the patient is connected to both the aorta and the pulmonary artery. **d** 3D-printed model of a coronary fistula. A giant right coronary artery fistula drained to the left ventricle is shown. AAo: ascending aorta, CAF: coronary artery fistula, CoA: coarctation of the aorta, DAo: descending aorta, Ao: aorta, LAA: left atrial appendage, LA: left atrium, LV: left ventricle, RA: right atrium, RV: right ventricle. Image data, 3D computer reconstructions, and 3D-printed models are from the Department of Cardiovascular Surgery, Xijing Hospital

With the help of 3D models, the surgical staff of the Department of Cardiovascular Surgery at Xijing Hospital has performed more than 100 surgical procedures for patients with complex CHDs including pulmonary atresia, complete transposition of the great arteries, double outlet right ventricle, cor triatriatum, coarctation or disconnection of the aorta, vascular ring, abnormal coronary artery, and persistent truncus arteriosus (Fig. 3). The models have helped the medical professionals to understand the internal spatial structure of the complex CHD and to formulate individual plans for surgical procedures. The operating time was greatly shortened, and the outcomes were good. The 3D-printed models were valuable for decision-making and intraoperative orientation.

**Fig. 4:**
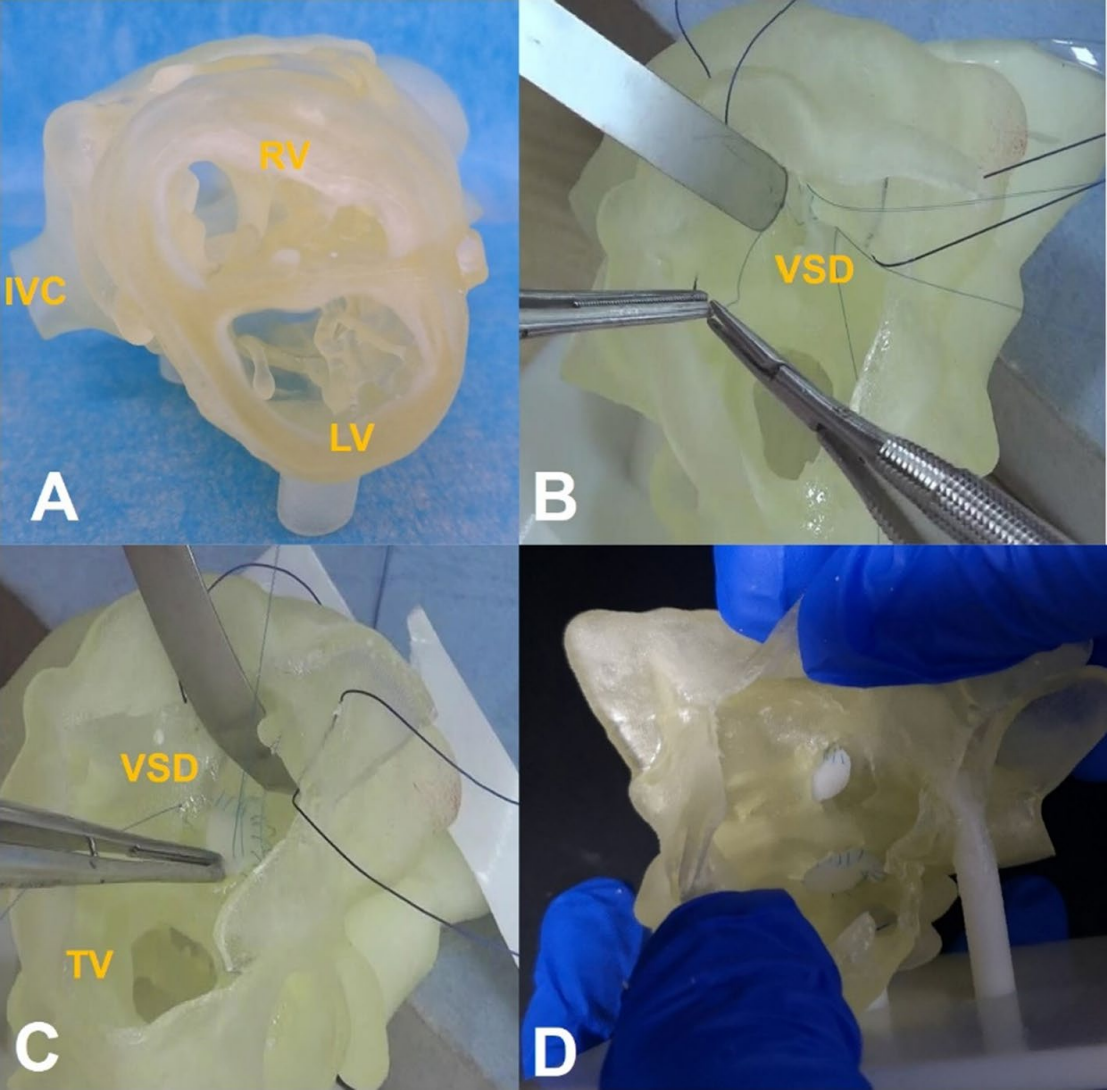
Three-dimensional (3D)-printed models of congenital heart diseases used for training. **a** 3D-printed model of a ventricular septal defect. **b** Training for the basic operative skills of suturing using a 3D-printed model. **c** Training young doctors and medical students using a 3D-printed model. **d** Evaluation of the suture technique using a 3D-printed model. VSD: ventricular septal defect, IVC: inferior vena cava, LV: left ventricle, RV: right ventricle, TV: tricuspid valve. The 3D-printed models are from the Department of Cardiovascular Surgery, West China Hospital

Doctors from the Department of Cardiovascular Surgery, West China Hospital, used 3D-printed models to mimic cardiac surgical procedures, thereby teaching and training young doctors and medical students. 3D models were printed before the operations, and the medical professionals could perform resections, suturing, and retraction and could simulate key steps of the operation using the individualized models (Fig. 4).

**Fig. 5:**
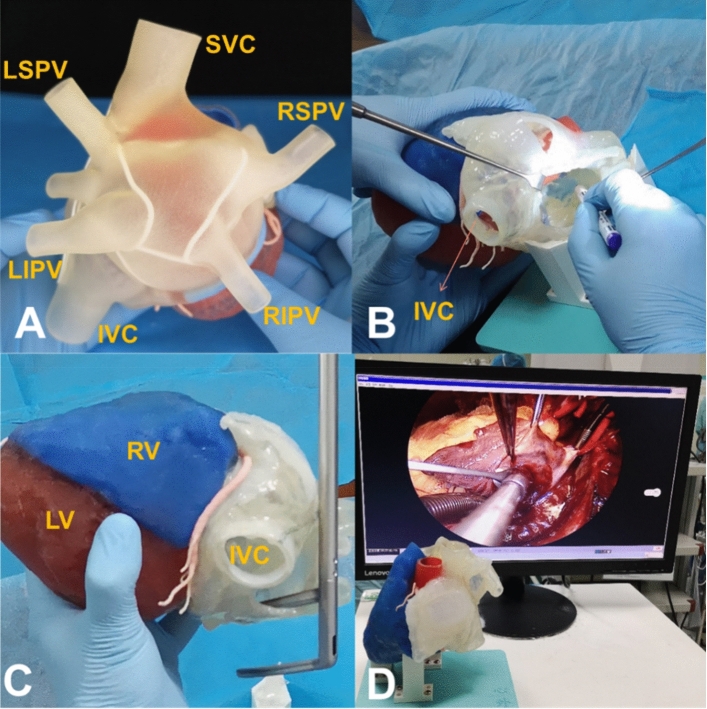
Three-dimensional (3D)-printed heart model to mimic the radiofrequency ablation of atrial fibrillation and a mitral valve repair operation in a patient with mirror-image dextrocardia. **a** 3D-printed heart model with planned ablation route; **b** surgical planning of the ablation route; **c** using the atrial fibrillation clamp to mimic the ablation procedure; **d** 3D printing technique-guided radiofrequency ablation of atrial fibrillation and mitral valve repair operation. IVC: inferior vena cava, LSPV: left superior pulmonary vein, LIPV: left inferior pulmonary vein, LV: left ventricle, RIPV: right inferior pulmonary vein, RSPV: right superior pulmonary vein, RV: right ventricle, SVC: superior vena cava. Image data, 3D computer reconstruction, and 3D-printed model are from the Department of Cardiovascular Surgery, Xiangya Second Hospital of Central South University

Doctors from the Department of Cardiovascular Surgery, Xiangya Second Hospital of Central South University, used the 3D-printed heart model to mimic radiofrequency ablation of atrial fibrillation and mitral valve repair in a patient with mirror-image dextrocardia. By repeatedly practicing on the 3D model, the medical team became familiar with the special anatomical structure of the patient, which increased confidence and strengthened collaboration among the team members. The special rare case was successfully completed with a shortened procedural time and no complications (Fig. 5).

## Three-dimensional printing and transcatheter aortic valve replacement

Transcatheter aortic valve replacement (TAVR) is an emerging minimally invasive valve replacement operation to treat severe aortic valve disease. This type of operation does not require cardiopulmonary bypass and results in rapid postoperative recovery [32, 33]. Since its introduction, TAVR has been increasingly used and has benefited more than 500,000 patients [34, 35]. The effectiveness and safety of TAVR have been widely recognized in recent years, and the procedure was approved in 2020 by the U.S. Food and Drug Administration to use in low-risk patients with aortic stenosis. Although the TAVR operation is simpler than the traditional surgical procedure, its risks and complications should not be underestimated. Familiarity with and accurate positioning of the instruments comprise the core of the TAVR operation. Unlike that of traditional surgical procedures, the success of TAVR depends more on devices and imaging. Preprocedural evaluation, strategic planning, and complication prevention are of vital importance in the success of TAVR. 3D printing technology, as an emerging useful tool, plays a more and more important role in improving the success rate of TAVR [36–39].

### Mimicking the transcatheter aortic valve replacement procedure

**Fig. 6:**
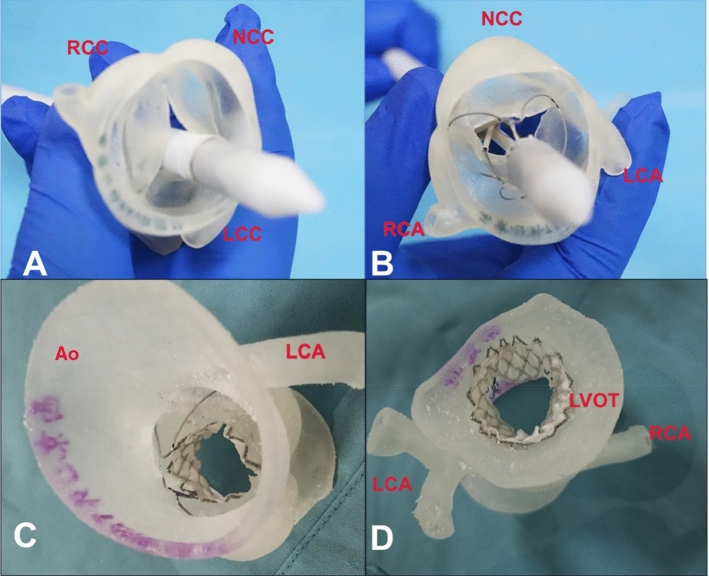
Three-dimensional (3D)-printed heart model to mimic the transcatheter aortic valve replacement (TAVR) procedure. **a** Transducing system across the valve; **b** releasing the step 1 button; **c** fully releasing the stented valve; **d** left ventricle view of the model to evaluate the results of the TAVR procedure. Ao: aorta, LCA: left coronary artery, LCC: left coronary cusp, LVOT: left ventricular outflow tract, NCC: non-coronary cup, RCA: right coronary artery, RCC: right coronary cups. Image data, 3D computer reconstruction, and 3D-printed model are from the Department of Cardiovascular Surgery, Xijing Hospital

After nearly 20 years of development, approximately 50 kinds of devices are used in TAVR procedures worldwide. Many of them involve multiple steps and finely tuned operative procedures, and one careless move can have serious adverse consequences. Therefore, adequate training is needed to abbreviate the TAVR learning curve and to improve procedural safety and clinical success. Using the 3D model, the TAVR operation can be simulated in vitro, which helps the surgeon and the heart team become more familiar with the performance of the different TAVR devices, shortens the learning curve, improves the technical success rate of the team, and reduces mortality and complications among the patients. Figure 6 illustrates how to mimic the transapical TAVR procedure using a 3D-printed model and a J-valve system (Jiecheng Ltd., Suzhou, China). Due to the complexity of the system, physicians benefit from practicing and becoming familiar with each step of the releasing mechanism of the device, further improving the outcome of the TAVR procedure.

### Predicting the possibility of conduction block

Postoperative conduction block may be attributed to calcified plaque displacement, which causes sustained pressure on or permanent damage to the atrioventricular conduction system at the junction of the right coronary valve and the non-coronary valve. The ventricular end of the prosthetic valve may also damage the conduction system located at the ventricular septum. Researchers from the Georgia Institute of Technology and the Piedmont Heart Institute printed out a model of aortic stenosis with calcification and simulated the process of balloon dilation and valve releasing [10]. The researchers were able to detect the direction of the deviation and observe the orientation of the stent valve affected by severe calcification, thus evaluating the probability of conduction block. The results suggested that severe calcification of the root and annulus can easily cause the valve to shift laterally, thereby causing conduction block. Through in vitro simulation and comparison of different valves, the study also found that the shorter valve rack may cause less conduction block.

### Preventing coronary artery occlusion

Coronary artery occlusion after TAVR is a rare but extremely serious complication [40]. The mortality rate of coronary artery occlusion after TAVR may be > 50%. The risk factors for acute or delayed coronary artery occlusion include (1) large calcification at the edge of the left and right coronary sinuses; (2) coronary height < 10 mm; (3) sinus diameter < 30 mm or a small aortic sinus; and (4) long aortic valve leaflets. It is extremely important to accurately predict the dynamic changes of the anatomical structure before beginning the operation.

**Fig. 7:**
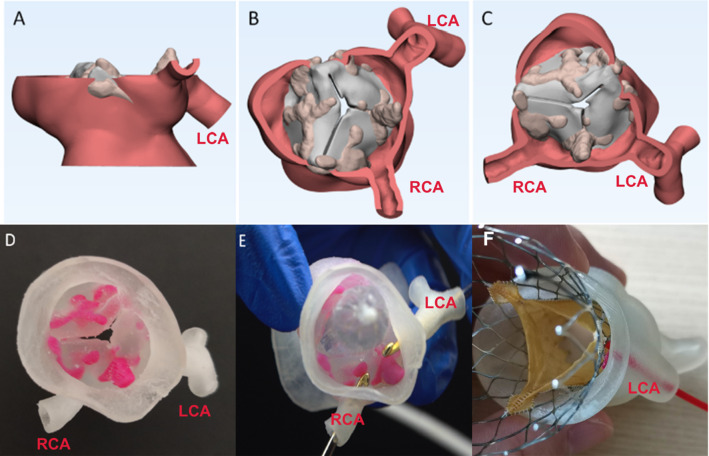
Three-dimensional (3D) digital model to evaluate the risk of coronary artery occlusion during TAVR. **a**–**c** Computer 3D model shows the calcification of the valve leaflets and the height of the coronary artery in patients at risk of coronary artery occlusion. **d** 3D-printed model shows the calcification of the valve leaflets and the height of the coronary artery. **e** In vitro balloon dilation using the 3D-printed model to evaluate the possibility of coronary obstruction.** f** In vitro valve implantation using the 3D-printed model to evaluate the possibility of coronary obstruction. LCA: left coronary artery, RCA: right coronary artery. Image data, 3D computer reconstruction, and 3D-printed model are from the Department of Cardiovascular Surgery, Xijing Hospital

In the Department of Cardiovascular Surgery, Xijing Hospital, doctors used the 3D-printed TAVR model to simulate the process of balloon dilation and the valve-releasing procedure to evaluate the possibility of coronary artery occlusion during TAVR and delayed coronary artery occlusion after surgery. If the patient is really at high risk, in vitro simulation would be helpful for the doctors to determine whether open surgery or a coronary-protecting strategy is indicated (Fig. 7).

### Predicting paravalvular leakage

Prediction of paravalvular leakage before TAVR is of great significance for developing a surgical strategy and selecting a valve. Researchers from the Georgia Institute of Technology and the Piedmont Heart Institute printed a multimaterial model of the aortic root [10]. By controlling the “diameter and bending wavelength” of the printed material, the model was able to simulate the physiological characteristics of the aortic tissue. The model can even show the special conditions of valve leaflets, such as calcification deposition and leaflet thickening, and depict the anatomical information accurately and completely. The results suggest that the 3D model can show exactly which patients are likely to develop paravalvular leakage and even the location and severity of the complication. Qian et al. established a model to quantitatively predict postoperative leakage after TAVR using 3D printing technology, which has shown great value in predicting the incidence, severity, and location of paravalvular leakage after TAVR [37].

## Three-dimensional printing and left atrial appendage occlusion

**Fig. 8:**
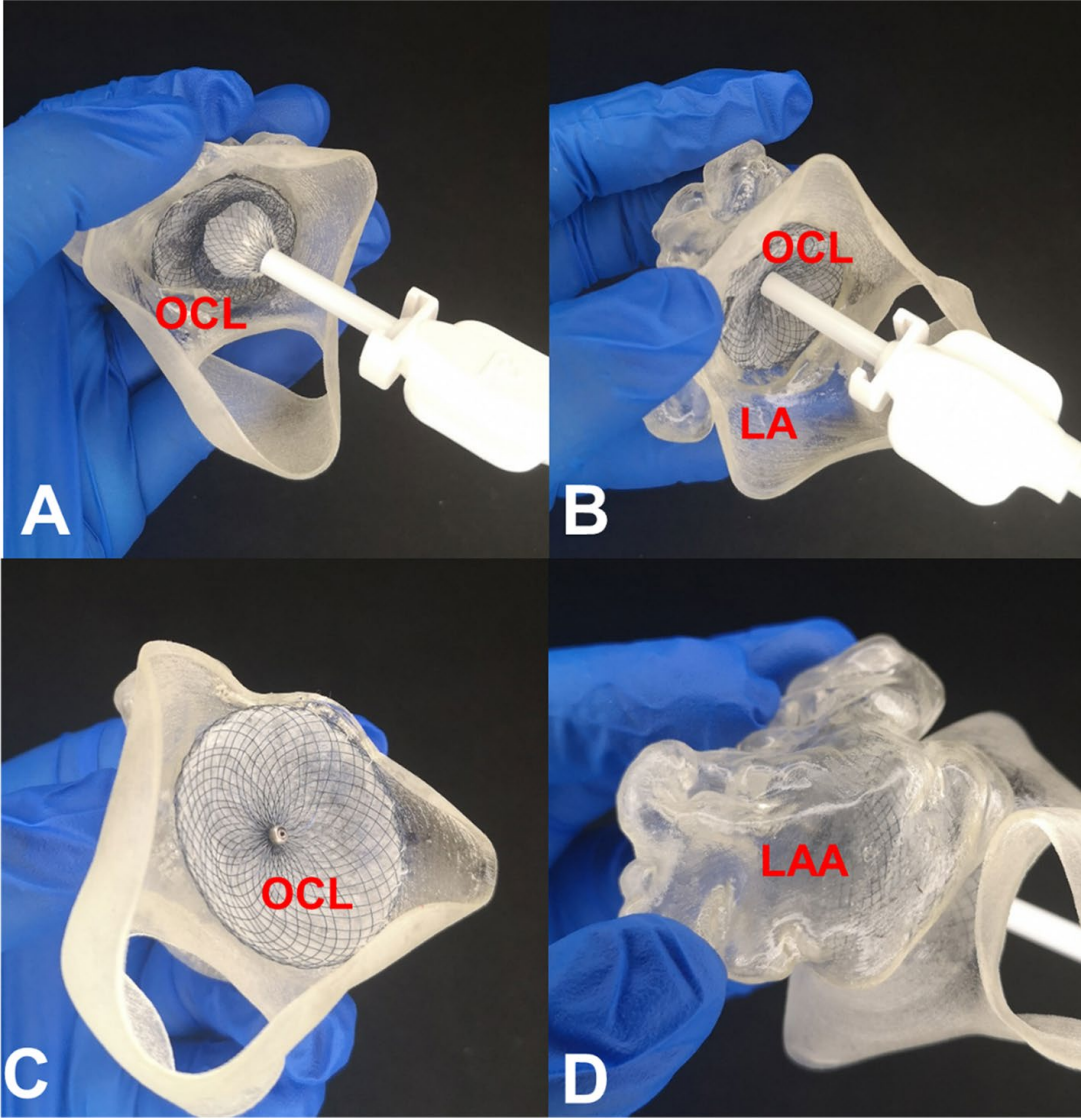
Three-dimensional (3D)-printed simulated left atrial appendage occlusion. **a** Partially released device using the 3D-printed left atrial appendage model; **b** push–pull experiment to test the stability of the device, **c** fully released device using the 3D-printed left atrial appendage model, **d** left atrial appendage view of the model to evaluate the results of the procedure. LAA: left atrial appendage, LA: left atrium, OCL: occluder. Image data, 3D computer reconstruction, and 3D-printed model are from the Department of Cardiovascular Surgery, the First Affiliated Hospital of Xi’an Jiaotong University

Transcatheter LAAO is a new method to prevent thromboembolism in patients with non-valvular atrial fibrillation by fixing the occluder inside the left atrial appendage (LAA) to prevent blood from entering the LAA. Its clinical benefits and safety have been confirmed by many clinical studies, and it has become an alternative treatment to prevent stroke in patients with atrial fibrillation. At the same time, the complexity and variability of LAA make it difficult to place the occluding devices in suitable locations. The occluder often needs to be replaced many times, which may increase the risk of LAA injury, thrombosis, and pericardial tamponade. In 2015, Otton et al. reported the successful treatment of LAAO in a 74-year-old patient, guided by 3D printing [41]. Transesophageal echocardiography examination showed that the diameter of the LAA opening was 15 and 18 mm. The LAA model was printed according to the data obtained from CTA. Three types of occluders, 21, 24, and 27 mm, were selected for test occlusion in vitro. They found that the 21-mm and the 27-mm occluders deployed in the 3D-printed model were either too small or too large; the 24-mm occluder was finally used during the operation to achieve complete occlusion. Currently, 3D printing technology can effectively assist decision-making related to LAAO [42–44]. Scholars of the First Affiliated Hospital of Xi’an Jiaotong University use 3D-printed models to simulate LAAO and to choose the appropriate size and position, using the push–pull experiment to test the stability of the device (Fig. 8).

## Three-dimensional printing and hypertrophic cardiomyopathy

Hypertrophic cardiomyopathy is a common inherited cardiac disease that is characterized by abnormal heart structure, asymmetrical cardiac hypertrophy, heart failure, and arrhythmia [45]. Current treatment methods mainly include drug therapy, surgical treatment, and alcohol septal ablation. Surgical treatment requires thoracotomy, which may result in a left bundle branch block, whereas alcohol ablation is less invasive but does not relieve obstruction of the left ventricular outflow tract in some patients [46, 47].

**Fig. 9:**
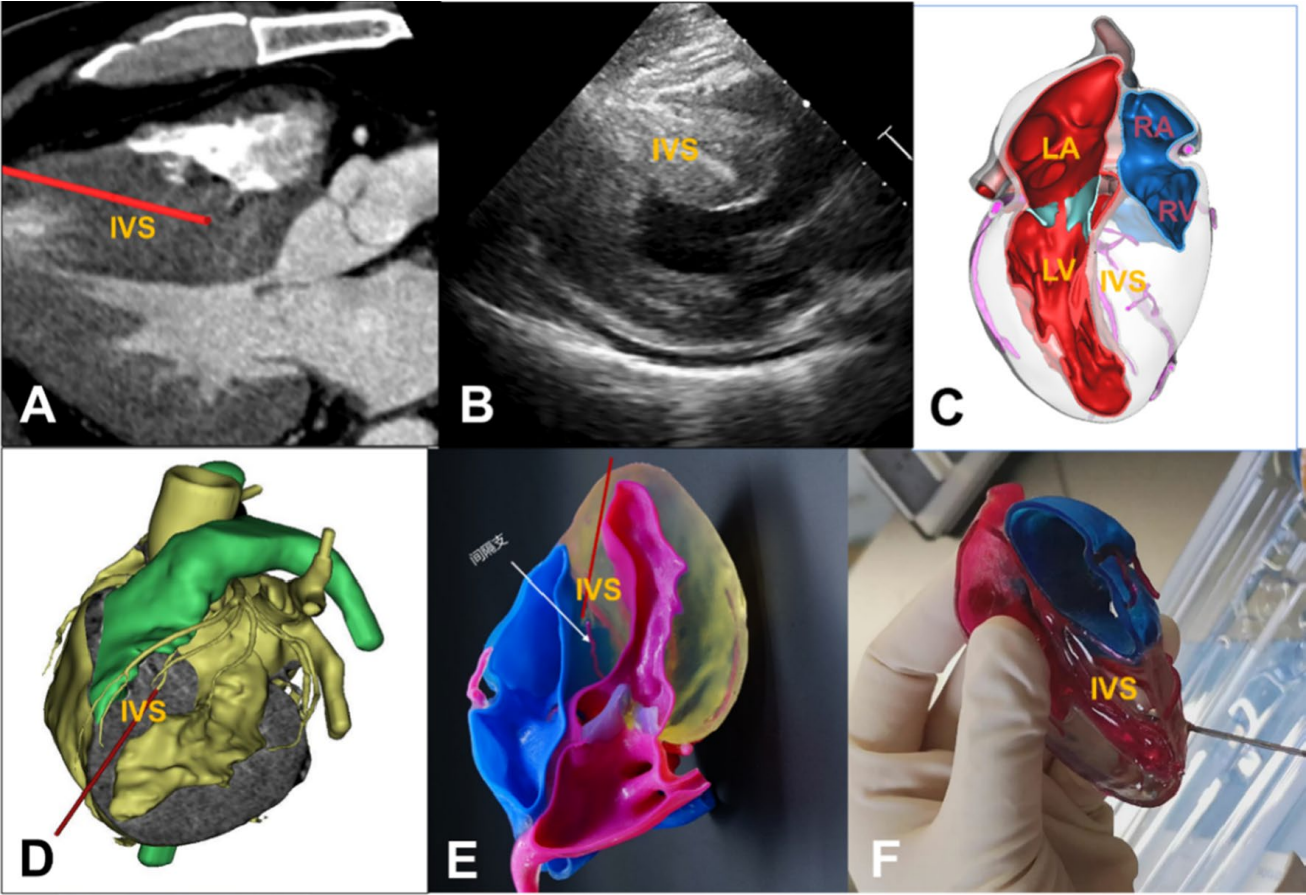
Ultrasound-guided percutaneous transluminal myocardial septal radiofrequency ablation (Liwen procedure) for the treatment of obstructive hypertrophic cardiomyopathy. **a** Computed tomography angiography (CTA) two-dimensional imaging display of the hypertrophic septum and the surgical path of radiofrequency ablation. The red line is a simulated electroacupuncture approach. The approach is shown to avoid the surface coronary artery and to ablate the septal branches during the operation. **b** Ultrasound images display the hypertrophic septum; the computer section shows the myocardium. **c** Digital three-dimensional (3D) model based on CTA images of the hypertrophic septum and the inner structure of the heart. **d** Digital 3D model based on CTA image display of the hypertrophic septum and the outer structure of the heart. **e** 3D cardiac printed model based on CTA images of the hypertrophic septum and the coronary artery branch. **f** Determination of the optimal puncture site and the proper electrode needle path using the 3D model. IVS: interventricular septum, LA: left atrium, LV: left ventricle, RA: right atrium, RV: right ventricle. Image data, computer 3D reconstruction, and 3D-printed models from the National Innovation Center for Additive Materials Manufacturing and the Department of Cardiovascular Surgery, Xijing Hospital

Dr. Liu Liwen, from the Department of Ultrasound, Xijing Hospital, has developed the Liwen procedure for hypertrophic obstructive cardiomyopathy, that is, percutaneous transmyocardial radiofrequency ablation under the guidance of ultrasound [48–50]. It is guided by ultrasound through a percutaneous transepicardial puncture through the tip of the heart to the area affected by ventricular septal hypertrophy; high-frequency waves can create local hypertrophic myocardial coagulation necrosis to achieve the goal of widening the left ventricular outflow tract. The 3D printing technique can be used to observe the anatomy of the heart, especially that of the distribution of the coronary arteries, and to guide the surgical planning for and the risk assessment of the Liwen procedure. A real 3D model of the heart was constructed using preoperative CTA or MRI data of patients with obstructive hypertrophic cardiomyopathy. The coronary artery was displayed in a computer 3D model or a 3D-printed model. To prevent the electrode from damaging the coronary artery and the conduction bundle and to ensure the safety of the operation, the anatomical structure of the patient's heart can be fully understood, the optimal puncture site can be determined, and the proper electrode needle path can be set (Fig. 9). At the same time, a 3D model corresponding to the CTA two-dimensional image and the ultrasound image can effectively guide the operation and shorten the operating time.

## Limitations

Several factors limit the current wide clinical application of 3D printing in cardiac medicine. First, the model is usually printed during a single phase of the cardiac cycle, which limits the accurate representation of the dynamic heart function. Second, the currently available printing materials cannot truly replicate the physical and mechanical properties of human cardiovascular tissue in terms of several physical parameters, such as steadiness, tensile strength, elasticity, and memory capacity. Third, lack of robust data and standardization, lack of an accepted method to assess the accuracy of the produced models, and lack of large-scale studies and randomized clinical trials, all limit the wide promotion of cardiovascular 3D printing techniques. Fourth, image segmentation, computer-aided design, printing, and postprinting preparation for clinical use all require a strong and efficient team whose members have abundant anatomical knowledge and passion, which is hard to establish in the circumstances caused by the coronavirus disease 2019 pandemic. Last but not least, the cost-effectiveness and timeliness of this additive technology, the complexity of the workflow, and the lack of uniform financing models and of reimbursement structures are key issues making this technology currently mostly limited to teaching hospitals and research institutions. To solve these problems, we should choose the optimal phase of the cardiac cycle for 3D printing on the basis of the specific case under consideration; develop novel printing materials to better replicate the physical structure of the cardiovascular anatomy; teach and train young doctors and medical students to be familiar with the workflow of 3D printing; carry out large clinical trials to illustrate the advantages and disadvantages of 3D printing; and reduce the cost and price of 3D printing, therefore promoting its clinical application and benefiting more patients.

## Future perspectives

With the rapid development of imaging and computer technology, the accuracy and simulation levels of cardiovascular 3D printing are constantly improving. 3D printing makes up for the inability of traditional image technology to precisely display complex structures, and computer 3D reconstruction helps cardiovascular physicians understand the pathological changes of human tissues more intuitively, which provides a new method for the precise treatment of patients with heart disease. Although the application of 3D printing in the cardiac field is still in its early stages, it can provide medical staff with in vitro visualization information and print individual 3D models to help doctors determine surgical procedures and promote communication between doctors and patients. It can also simulate surgical and interventional procedures, improve the success rate and safety of such procedures, and help train medical professionals and medical students. Meanwhile, new cardiovascular therapies might be developed based on 3D-printed models. With the further development of 3D printing technology, the advantages of customized and individualized manufacturing will help people to better understand cardiac diseases. In the future era of 3D+, developments in biological engineering technology, materials science, biology, computer science, and other disciplines will lead to the design of materials that can be implanted in the human body and result in new breakthroughs in the treatment of cardiac diseases. Meanwhile, the tissues and organs generated by biological 3D printing might also be applied in the treatment of cardiac diseases. 3D printing will constantly change the traditional treatment methods, improve the diagnosis and treatment of cardiac diseases, and better benefit patients with cardiac diseases.
